# A Single Dose of Methylprednisolone Improves Aversive Memory Consolidation and Extinction in Rats

**DOI:** 10.3389/fnins.2019.01167

**Published:** 2019-10-31

**Authors:** Rithiele Gonçalves, Liane da Silva de Vargas, Pâmela B. Mello-Carpes

**Affiliations:** Physiology Research Group, Federal University of Pampa, Uruguaiana, Brazil

**Keywords:** aversive memory, inhibitory avoidance, corticoids, memory persistence, memory extinction

## Abstract

Aversive memory is essential for survival, but in some situations its exacerbation can be potentially dangerous. There are several ways to modulate memory, among them, through stress-related hormones physiological release or administration of exogenous substances analogous to them. Recently, our group shown that a chronic treatment with a low dose of methylprednisolone (MP) is able to promote memory persistence in rats. Herein, we evaluate if a single intraperitoneal (IP) dose of MP (5 mg/kg) is able to modulate aversive memory consolidation and promote memory persistence and extinction in rats. For this, two experiments were carried out. In the first one, we demonstrated that a single IP MP administration in specific times after inhibitory avoidance (IA) training improved memory consolidation and persistence. In the second experiment, we verified that a single IP MP administration 2 h after IA extinction training promoted memory extinction. This results suggest a possible new clinical applicability for MP on the aversive memory disorders, as post-traumatic stress.

## Introduction

The aversive memory corresponds to our ability to identify dangerous situations when certain trigger stimuli promotes the retrieval of memories related to a prior fear experience. These memories are fundamental to activate circuits related to survival and necessary to avoid harmful circumstances to the individual (Ozawa et al., 2017). In this sense, the consolidation and persistence of this type of memory is essential to evolution and survival (Do Monte et al., 2016).

On the other hand, a strong fear memory can generate exacerbated responses to non-harmful stimulus (Keller et al., 2015), as observed in the Post-Traumatic Stress Disorder (PTSD) (Bisson et al., 2015). The extinction of fear memories has been widely used in the clinic as a form of treatment of PTSD. PTSD patients often remember their traumatic experiences over and over again, intensively and out of context, which can be severely disabling (Sher, 2010; Davis, 2011; Milad and Quirk, 2012; Furini et al., 2014). In exposure therapy, the extinction is used in a way by which the individuals are exposed to stimuli related to the one that have led them to a traumatic experience until they suppress the inadequate responses upon perceiving the absence of danger, becoming able to lead a normal life (Hendriks et al., 2018). In this way, the extinction is considered the formation of a new memory without erasing the original memory, but overlapping it (de Carvalho Myskiw et al., 2015). It is characterized by a progressive decrease in the intensity and/or frequency of the conditioned response caused by the repeated memory evocation in the absence of the unconditioned stimulus (Cammarota et al., 2005).

Although the extinction has been used in exposure therapy, its efficiency is limited (Maren and Holmes, 2016). In this way, several strategies and different protocols have been studied trying to promote and/or improve memory extinction. Among them is the use of drugs that modulate extinction, such as serotonergic and noradrenergic drugs, neuropeptides, endocannabinoids, glucocorticoids, and histone deacetylase inhibitors (Fitzgerald et al., 2014). Memory consolidation requires the synthesis of new proteins in the hippocampus, a process that can be affected by the use of some substances or by the endogenous release of others, such as hormones (Kandel and Schwartz, 1982; Carter et al., 2005; Gold, 2008). Glucocorticoids have been reported in the literature as important modulators of mnemonic responses (Buss et al., 2004; Brunner et al., 2005).

Methylprednisolone (MP) is a glucocorticoid commonly used in the therapy of allergies, inflammations and autoimmune disorders (Longui, 2007; Kajiyama et al., 2010). The effects of MP on memory have also been studied. Zhang et al. (2011) demonstrated that MP may causes memory impairment by causing deficits in synaptic plasticity in the hippocampus. On the other hand, a single corticosterone injection after training in a fear conditioning task seems to improve memory consolidation (Abrari et al., 2009). Additionally, our group demonstrated, in previously experiments carried out with rats, that the chronic treatment (10 days) with a low dose of MP (5 mg/kg), but not with a high dose (30 mg/kg), promotes aversive memory persistence, which was correlated to the improvement in long-term potentiation (LTP) (de Vargas et al., 2017). The contradictions found in the MP effects by the various authors can be attributed to the diversity of effects caused by glucocorticoids depending on the different temporal administration or the different doses used in each study. So, it is important developing studies on the effects of MP considering this factors – time of administration and dose. Additionally, most of the studies about MP effects investigates memory consolidation – to study the effects of MP on memory extinction is important, considering its potential clinical application.

Based on our previous results, here we aim to investigate the effects of a single MP dose on aversive memory consolidation and persistence, as well as on the extinction of an aversive memory. Therefore, we demonstrated in this study that a single dose of MP administrated in a low dose and in a specific temporal window, improves the consolidation and extinction of an aversive memory in rats, promoting the persistence of these events. These data set helps to suggest a possible new clinical applicability for MP on the aversive memory disorders, as PTSD and other conditions related to fear memories, as phobias.

## Materials and Methods

### Experimental Design

**FIGURE 1:**
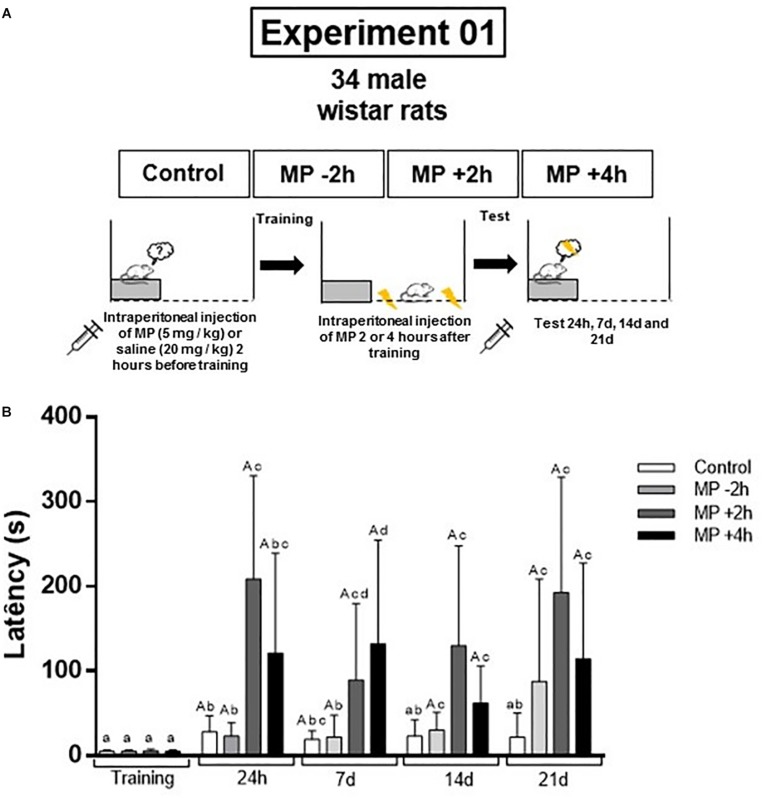
**(A)** Design of the experiment 1. The animals were divided in four groups (control group, that received saline solution, and MP groups, that received Methylprednisolone, 5 mg/kg, in different times before or after memory training; *n* = 8–9/group), and were trained in the inhibitory avoidance and tested 24 h, 7, 14, and 21 days after to evaluate memory consolidation and persistence. **(B)** A single dose of MP enhances the memory consolidation (24 h test) and promotes the memory persistence (7, 14, and 21 days tests). Different letters indicate difference between training and test and/or between groups (*P* < 0.05; Wilcoxon test or two-way ANOVA followed by Sidak’s *post hoc* were used; data are expressed as median ± SEM). MP = Methylprednisolone.

**FIGURE 2:**
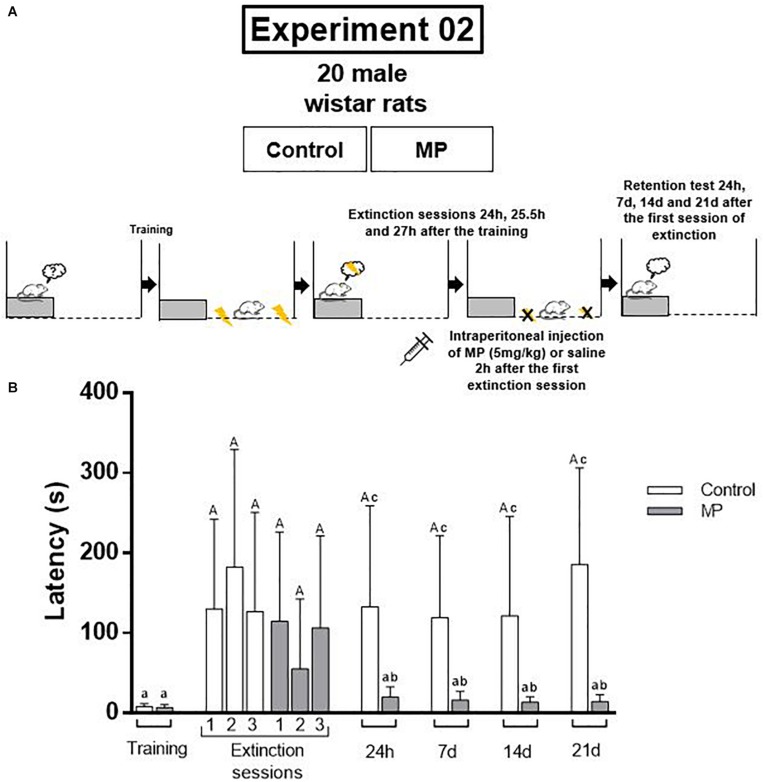
**(A)** Design of the experiment 2. The animals were divided into two groups (control received saline and MP received methylprednisolone 5 mg/kg, *n* = 10/group), and were trained in inhibitory avoidance task. In the following day, the animals were submitted to three extinction sessions. The retention and persistence of extinction memory were tested 24 h, 7, 14, and 21 days after the first extinction session. **(B)** MP promotes the extinction of aversive memory and its persistence for 21 days. Different letters indicate difference between training and test and/or between groups (*P* < 0.05; Wilcoxon test or Mann–Whitney test were used; data are expressed as median ± SEM). MP = Methylprednisolone.

This study was composed by two experiments. The first one was carried out in order to verify if a single dose of MP is able to improve the aversive memory consolidation, promoting its persistence, as well as to determine the optimal time for drug administration. For this, the animals were divided in four groups (control group, that received saline solution, and MP groups, that received Methylprednisolone, 5 mg/kg; *n* = 8–9/group), and were trained in the inhibitory avoidance (IA) and tested 24 h, 7, 14, and 21 days after to evaluate memory consolidation and persistence (Figure 1A). The second experiment was performed considering the results of the first one, in order to verify the effects of this single dose of MP on the aversive memory extinction. For this, the animals were divided into two groups (control received saline and MP received methylprednisolone 5 mg/kg, *n* = 10/group), and were trained in IA task. In the following day, the animals were submitted to three extinction sessions. The retention and persistence of extinction memory were tested 24 h, 7, 14, and 21 days after the first extinction session (Figure 2A).

### Animals

Fifty-four adult male Wistar rats (3 months old, 250–280 g) were purchased from the Central Vivarium of Federal University of Santa Maria (RS/Brazil) and housed four per cage under controlled light and environmental conditions (12 h light/12 h dark cycle at 23 ± 2°C and 50 ± 10% humidity) with food and water *ad libitum*. The study was performed according to and approved by the Ethics Committee for Animal Use from Federal University of Pampa/Brazil (protocol #20/2016).

### Drugs

Methylprednisolone sodium succinate (MP, Solu-Medrol^®^) was purchased from Pfizer (São Paulo, SP, Brazil). The treated animals received a single dose of MP (5 mg/kg) dissolved in saline and administered intraperitoneally according to the group treatment (2 h before or after training and 4 h after training; i.p.). The dose was defined based on previous studies (de Vargas et al., 2017). The control groups received saline (vehicle; i.p). The temporal window of the drug administration is based on studies (Hall et al., 1984; Bannwarth et al., 1997).

### Experimental Protocols

#### Inhibitory Avoidance Learning

Inhibitory avoidance is a commonly behavioral task used to investigate learning and memory processes in rodents (Gold, 1986; McGaugh and Roozendaal, 2009). The device used in this study consisted in a 50 × 25 × 50 cm metal box, with a transparent acrylic made front. The floor of the apparatus consisted of an array of parallel electrified bars with a platform box of 5.0 cm height by 7.0 cm wide placed in the left side of the box. During IA training, rats were carefully placed on the elevated platform and they receive a single aversive foot shock (0.5 mA for 2 s) when stepping down from the platform and all four legs contacted with the electrified bars (Rossato et al., 2009). After this, the animals were immediately placed into their housing-boxes. Retention of the training was tested at 24 h and then every week for the 3 weeks after training by measuring rats’ latency to step down from the platform. The cut-off time for the IA was 300 s. Longer retention test latencies (compared to training ones) are interpreted as good memory consolidation and/or persistence of memory.

#### IA Extinction Learning

The aversive memory extinction was investigated in the second experiment (Figure 2A). The animals were submitted to non-reinforced sessions different times after training: 24, 25.5, and, 27 h (Cammarota et al., 2005). At each extinction session, the animals were placed on the training box platform until they stept down with all four feet on the floor. No shock was given and the animals could freely explore the box for 30 s before being returned to their housing-boxes. In order to evaluate the extinction of aversive memory, a retention test was performed 24 h after the last extinction session. The persistence of aversive extinction memory was evaluated in tests performed every week for 3 weeks.

#### Control Behavioral Tasks

All animals were subjected to control behavioral experiments to avoid biases of the effect of MP injection on other behavioral parameters that could influence the IA learning and alter the tests results. The control behavior tests were performed in each test day in both experiments (1 and 2). The exploratory and locomotor behaviors were analyzed in the Open Field (OF) test, in which crossings and rearings of each rat were monitored over 5 min (Bonini et al., 2006). In order to evaluate anxiety-like behavior, rats were exposed to an elevated plus maze (EPM). The time spent and the total number of entries into the open and closed arms were recorded over a 5 min session (Pellow et al., 1985). Finally, to ensure that periphery sensitivity did not change upon MP administration, we recorded the latency time for tail removal in the tail flick (TF) test (Tjolsen et al., 1989).

### Statistical Analysis

First of all, the Shapiro–Wilk test was used to check the data distribution. After this, statistical analysis of data from IA was performed using a Wilcoxon test for intragroup comparison (training vs. test), and two-way ANOVA test followed by Sidak’s *post hoc* (to compare more than two groups, in the case of the first experiment) or Mann-Whitney (to compare two groups, in the case of the second experiment) were used for comparisons between the different groups. These data were present as median ± SEM. The OF, EPM and TF results were analized by two-way ANOVA. These data were present as mean ± SEM. Values of *P* ≤ 0.05 were considered significant in all cases.

## Results

### Experiment 1 – Effects of a Single Dose of MP (5 mg/kg) in Aversive Memory Consolidation and Persistence

In the first experiment we could verify that all the groups that received MP administration after IA training improved the memory consolidation and persistence (Figure 1B).

In the 24 h test, all groups showed increased step-down latencies (*P* = 0.007 for control; *P* = 0.0002 for MP −2 h; *P* = 0.003 for MP +2 h; *P* = 0.01 for MP +4 h) when compared to their training sessions, demonstrating that all groups presented memory consolidation. The analyze of 24 h test latencies showed a main effect for treatment [*F*_(__1_,_42__)_ = 7332; *P* < 0.0001] and time [*F*_(__2_,_42__)_ = 2254; *P* < 0.0001], with significant interaction between the factors [*F*_(__2_,_42__)_ = 2254; *P* < 0.0001]. MP +2 h and MP +4 h groups presented higher step-down latency than control group (*P* < 0.0001).

In the 7 days test, all groups showed increased step-down latencies (*P* = 0.007 for control; *P* = 0.01 for MP −2 h; *P* = 0.003 for MP +2 h; *P* = 0.01 for MP +4 h) when compared to the training session. The analyze of 7 days test latencies showed a main effect for treatment [*F*_(__1_,_42__)_ = 18.27; *P* = 0.0001] and time [*F*_(__2_,_42__)_ = 7.117; *P* = 0.0022], with significant interaction between the factors [*F*_(__2_,_42__)_ = 7.117; *P* = 0.002]. MP +2 h and MP +4 h groups presented higher step-down latency than control group (*P* < 0.01).

In the 14 days test, only the MP groups showed increased step-down latencies (*P* = 0.07 for control; *P* = 0.04 for MP −2 h; *P* = 0.01 for MP +2 h; *P* = 0.01 for MP +4 h) when compared to the training session. The analyze of 14 days test latencies showed a main effect for treatment [*F*_(__1_,_42__)_ = 14.57; *P* = 0.004]. No effect of time were found [*F*_(__2_,_42__)_ = 0.9913; *P* = 0.3796], with no significant interaction between the factors [*F*_(__2_,_42__)_ = 0.9913; *P* = 0.3796). MP +2 h group presented higher step-down latency than control group (*P* < 0.01).

In the 21 days test, only the MP groups showed increased step-down latencies (*P* = 0.31 for control; *P* = 0.007 for MP −2 h; *P* = 0.01 for MP +2 h; *P* = 0.01 for MP +4 h) when compared to training session. The analyze of 21 days test latencies showed a main effect for treatment [*F*_(__1_,_42__)_ = 153.8; *P* < 0.0001] and time [*F*_(__2_,_42__)_ = 78.61; *P* < 0.0001], with significant interaction between the factors [*F*_(__2_,_42__)_ = 78.61; *P* < 0.0001]. MP +2 h (*P* < 0.0001) and MP +4 h (*P* < 0.05) groups presented higher step-down latency than control group.

### Experiment 2 – Effects of a Single Dose of MP (5 mg/kg) on Aversive Memory Extinction and Persistence of Memory Extinction

Considering the results of the first experiment, in the second experiment the time of MP administration used was 2 h after the extinction training. The results show that MP promotes the memory extinction (Figure 2B).

In the extinction sessions the rats of both groups (control and MP) remember the original memory (*P* < 0.05, training vs. each extinction session, Wilcoxon test). In the retention tests the original memory persisted in control group, despite of the extinction sessions (*P* = 0.03 for 24 h test; *P* = 0.03 for 7 days test; *P* = 0.01 for 14 days test; *P* = 0.01 for 21 days test; training vs. test, Wilcoxon test). On the other hand, the rats from MP group were able to extinguish the aversive memory (*P* = 0.12 for 24 h test; *P* = 0.06 for 7 days test; *P* = 0.07 for 14 days test; *P* = 0.15 for 21 days test; training vs. test, Wilcoxon test). Comparisons between groups in test days show differences in all tests (*P* = 0.03 on 24 h test; *P* = 0.008 for 7 days test; *P* = 0.0003 for 14 days test; *P* = 0.0006 for 21 days test), being the step-down latencies of MP group lower than control group.

### Control Behavioral Tasks

No significant differences were observed in the control behavioral tasks, demonstrating that the results observed on IA tests are related exclusively to MP effects on memory (*P* > 0.05; Table 1).

**TABLE 1:** Methylprednisolone did not alter locomotor and exploratory activity, anxiety, and periphery sensitivity of the animal.

**Behavioral task and parameter evaluated**		**Control**	**MP −2 h**	**MP +2 h**	**MP +4 h**
EPM	Total entries in the open arms (*n*)	1.75 (1.38)	3.77 (2.27)	3.44 (3.24)	3.00 (2.61)
	Time in open arms (*s*)	90.75 (31.96)	92.89 (55.41)	124.9 (60.97)	86.88 (32.68)
OF	Crossings (*n*)	41.63 (23.30)	52.89 (16.24)	50.00 (25.51)	51.63 (23.29)
	Rearings (*n*)	13.50 (8.12)	16.00 (6.36)	17.00 (7.03)	18.13 (8.91)
TF	Latency (*s*)	75.45 (5.58)	75.10 (7.09)	80.48 (7.80)	79.61 (7.90)

*Data are shown as mean ± SEM; *n* = 8–10/group; *P* > 0.05 for all comparisons (two-way ANOVA). EPM = elevated plus maze; MP = Methylprednisolone; OF = open field; TF = tail flick.*

## Discussion

Our results demonstrated that a single dose of MP administrated in an specific time after to memory acquisition is able to improve the aversive memory consolidation and to promote aversive memory extinction.

There are two main novelties in these results. The first one is the demonstration that even in a single dose MP is effective to promote memory modulation. Previously, our group show that chronic treatment with a low dose of MP promotes the persistence of aversive memory (de Vargas et al., 2017); therefore, MP is effective as a memory enhancement tool, both in acute and chronic administration. A single dose of MP promoted the persistence of the aversive memory for 21 days, unlike the control group, which naturally passes through a process of forgetting – best memory persistence was verified in administrations made after IA training. Some studies associated the acute effects of the glucocorticoids with the beneficial to memory consolidation and the strength of the memory (Joels et al., 2011; Sandi, 2011), but the dependence of the administration time and of the drug dose often make difficult to find the temporal window and dose to have the best effect, which in this study, we have succeeded.

The second important novelty demonstrated in our research is that a single dose of MP facilitates the aversive memory extinction. Only the animals that received MP were able to extinguish the aversive memory after the extinction sessions; and, importantly, this effect persisted for 21 days. Considering that aversive and fear memories are generally strong memories, extinction is a difficult process, and, although it is used in clinic for PTSD treatment, it represents a challenge for therapists. Thus, MP appears as a potential alternative to facilitate this process, since a single dose administrated after extinction training was able to facilitate the extinction learning.

In a study with patients with acrophobia, de Quervain et al. (2011) showed that a 20 mg dose of cortisol given orally 1 h prior to exposure therapy was able to facilitate extinguishing and promote it for up to 1 month. This study demonstrates the real clinical applicability of the results presented here, since MP is already a commercialized drug and it is a new possibility of use in the medical clinic. In this sense, in another study using cortisol as a pharmacological therapy to improve the exposure therapy results, United States veterans showed improved retention of extinction when used the associated therapy (Yehuda et al., 2015). The extinction and consolidation enhancement promoted by the glucocorticoids share some mechanisms. Some authors claim that glucocorticoids, when acting in a memory extinction window, would act to prevent the exacerbation of that fear response, facilitation the extinction learning and providing the subject with an adequate response to each situation (de Quervain et al., 2019).

Even though IA training consisting in a single trial, the brain processes underlying the task acquisition are complex. Rats must encode different pieces of information in order to acquire a correct association between a particular location within the apparatus and the aversive stimulus of foot shock, which involves the hippocampus, the amygdala and the prefrontal cortex. In this sense, the use of a single low dose of MP shows a new possibility as cognitive enhancer, since the persistence of memory is related to a series of events, as synaptic neuroplasticity, which are directly related to cognition (Takeuchi et al., 2014). Among these events, as demonstrated previously by our group and other authors, LTP and the biochemical events associated with it are necessary for the formation of a strong memory (Izquierdo et al., 2008; de Vargas et al., 2017).

Whitehead et al. (2013) verified that an acute stress in rats can enhance the LTP when compared an unstressed rats, and Liston et al. (2013) demonstrated that, acutely, glucocorticoids can promote long-term memory through a learning mechanism dependent of dendritic spines. Glucocorticoids, at different concentrations, act by modulating LTP in different ways, and at low concentrations could act in a signaling mechanism through BDNF, particularly increasing levels of TrkB receptors (Peters et al., 2018). The influence of MP on LTP was verified in our previously work (de Vargas et al., 2017). In another way, some studies have demonstrated that glucocorticoids are capable to potentiate glutamatergic transmission, acting improving post-synaptic traffic of AMPA and NMDA receptors, which would increase the activation of intracellular proteins fundamental for memory persistence, such as MAPK and CaMKII (Conboy and Sandi, 2010; Popoli et al., 2011; Sandi, 2011). Chen et al. (2012) showed that the consolidation of memory mediated by glucocorticoid receptors involves the CaMKIIα-BDNF-CREB pathway and culminates with increased expression of structural proteins such as Arc, fundamental for stabilization and synapses and consequently for long-term memory.

Thus, this study suggests a new clinical applicability for MP both as a cognitive enhancer for memory-impairing diseases, promoting memory persistence, as well as a facilitator of memory extinction and an auxiliary tool in the treatment of disorders such as PTSD. The administration of MP after learning or extinction session proved to be more effective. Anyway, MP probably share mechanisms of action with glucocorticoids, but new studies are necessary to better elucidate them.

## Data Availability Statement

The raw data supporting the conclusions of this manuscript will be made available by the authors, without undue reservation, to any qualified researcher.

## Ethics Statement

The study was performed according to and approved by the Ethics Committee for Animal Use from Federal University of Pampa/Brazil (protocol #20/2016).

## Author Contributions

RG and LV performed the experiments, analyzed the data, and wrote the manuscript. PM-C analyzed the data and wrote the manuscript.

## Conflict of Interest

The authors declare that the research was conducted in the absence of any commercial or financial relationships that could be construed as a potential conflict of interest.
